# Identifying Priority and “Bright-Spot” Counties for Diabetes Preventive Care in Appalachia: An Exploratory Analysis

**DOI:** 10.13023/jah.0101.04

**Published:** 2019-04-30

**Authors:** Peter J. Mallow, Michael Topmiller, Jennifer Rankin, Jene Grandmont, David Grolling, Jessica L. McCann, Mark Carrozza

**Affiliations:** Xavier University, Health Economics and Clinical Outcomes Research, mallowp@xavier.edu; American Academy of Family Physicians, HealthLandscape, mtopmiller@aafp.org; American Academy of Family Physicians, HealthLandscape, jrankin@aafp.org; American Academy of Family Physicians, HealthLandscape, jgrandmont@aafp.org; American Academy of Family Physicians, HealthLandscape, dgrolling@aafp.org

**Keywords:** Type 2 Diabetes Mellitus (T2DM), preventive care, conditional maps, bright spots, Appalachia

## Abstract

**Introduction:**

Type 2 diabetes mellitus (T2DM) prevalence and mortality in Appalachian counties is substantially higher when compared to non-Appalachian counties, although there is significant variation within Appalachia.

**Purpose:**

The objectives of this research were to identify low-performing (priority) and high-performing (bright spot) counties with respect to improving T2DM preventive care.

**Methods:**

Using data from the Centers for Medicare and Medicaid (CMS), the Dartmouth Atlas of Health Care, and the Appalachia Regional Commission, conditional maps were created using county-level estimates for T2DM prevalence, mortality, and annual hemoglobin A1c (HbA1c) testing rates. Priority counties were identified using the following criteria: top 33rd percentile for T2DM mortality; top 33rd percentile for T2DM prevalence; bottom 50th percentile for A1c testing rates. Bright spot counties were identified as counties in the bottom 33rd percentile for T2DM mortality, the top 33rd percentile for T2DM prevalence; and the top 50th percentile for HbA1c testing rates.

**Results:**

Forty-one priority counties were identified (those with high T2DM mortality, high T2DM prevalence, and low HbA1c testing rates), which were located primarily in Central and North Central Appalachia; and 17 bright spot counties were identified (high T2DM prevalence, low T2DM mortality, and high HbA1c testing rates), which were scattered throughout Appalachia. Eight of the 17 bright spot counties were adjacent to priority counties.

**Implications:**

By employing conditional mapping to T2DM, multiple variables can be summarized into a single, easily interpretable map. This could be valuable for T2DM-prevention programs seeking to prioritize diagnostic and intervention resources for the management of T2DM in Appalachia.

## INTRODUCTION

Type 2 diabetes mellitus (T2DM) often occurs due to obesity, and when it is not properly managed, it is a major driver of preventable hospitalizations and healthcare costs. The Appalachian Region, which consists of 420 counties across 13 states (Alabama, Georgia, Kentucky, Maryland, Mississippi, New York, North Carolina, Ohio, Pennsylvania, South Carolina, Tennessee, Virginia, and West Virginia) has higher rates of T2DM, possibly earlier onset of the disease, and worse health outcomes when compared to the rest of the U.S.^1^ T2DM prevalence in Appalachian counties is 21% higher and T2DM-related mortality is 11% higher when compared to non-Appalachian counties.^2^ Hemoglobin A1c (HbA1c) testing rates are higher in Appalachian states than in the non-Appalachian states,^3^ yet diabetic outcomes are worse.^1^,^2^ However, the Appalachian Region is not homogenous with respect to T2DM rates, HbA1c testing, and diabetic outcomes. While recent research from the Appalachian Regional Commission (ARC) presented an analytical framework for identifying high-performing counties within Appalachia based on several health measures, simpler approaches that focus on specific measures that can be replicated by state and local health departments are needed.^2^ This current research illustrates a conditional mapping technique for identifying low-performing (priority) and high-performing (bright-spot) counties with respect to improving T2DM preventive care.

## METHODS

Due to the heterogeneity of the population in Appalachian counties, an empirical Bayes approach was used to create county-level estimates for T2DM prevalence and annual HbA1c testing rates using data from the Centers for Medicare and Medicaid (CMS) and the Dartmouth Atlas of Health.^3^,^4^ The empirical Bayes approach smooths rates toward the overall average, based on total population. Thus, counties with low population numbers have their rates smoothed more toward the overall population average compared to counties with larger populations.^5^ The data included all fee-for-service Medicare beneficiaries. The Dartmouth data included fee-for-service beneficiaries aged 65–74 years with T2DM who received an HbA1c test. Other county level measures included age-adjusted T2DM mortality estimates (2008–2014), rural status from the Creating a Culture of Health in Appalachia project,^2^ and poverty rates and distressed economic status from the ARC. *Distressed* was defined as Appalachian counties that rank in the bottom 10% for the entire U.S. based on unemployment, per-capita market income, and poverty.^6^

**Figure 1:**
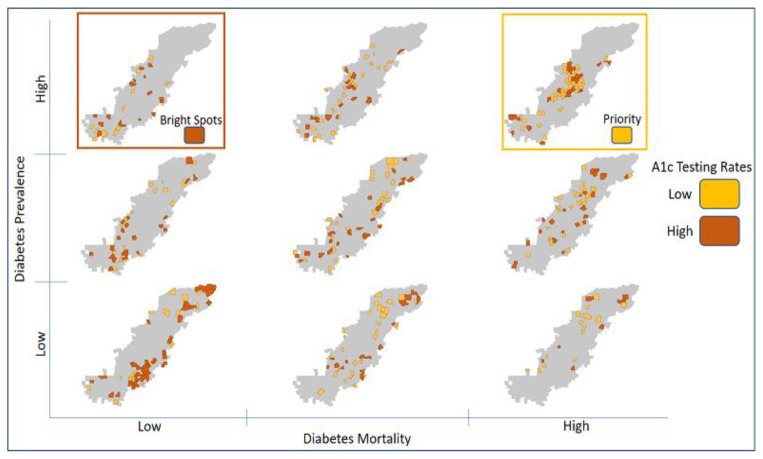
HbA1c testing rates, conditioned on diabetes prevalence and mortality

Conditional maps were created at the county level, which allowed for visualizing the relationship between three variables simultaneously.^7^ The variables included: rates of HbA1c testing, T2DM prevalence, and T2DM mortality. The resulting conditional map allows the examination of spatial patterns and were used to identify low-performing (priority) and high-performing (bright-spot) counties within Appalachia. HbA1c testing rates were mapped, conditioned on diabetes prevalence and mortality (Figure 1), which allowed the visualization of HbA1c testing rates for Appalachian counties across different categories based on their rates of T2DM mortality and prevalence. Priority counties can be found in the upper right corner of Map 1 (colored yellow–orange) and were identified using criteria based on the distribution of the datasets: counties in the top 33rd percentile for T2DM mortality; counties in the top 33rd percentile for T2DM prevalence; and counties in the bottom 50th percentile for HbA1c testing rates. Bright-spot counties can be found in upper left corner of Map 1 (colored brown) and were identified as counties in the bottom 33rd percentile for T2DM mortality, the top 33rd percentile for T2DM prevalence; and the top 50th percentile for HbA1c testing rates.

## RESULTS

**Figure 2:**
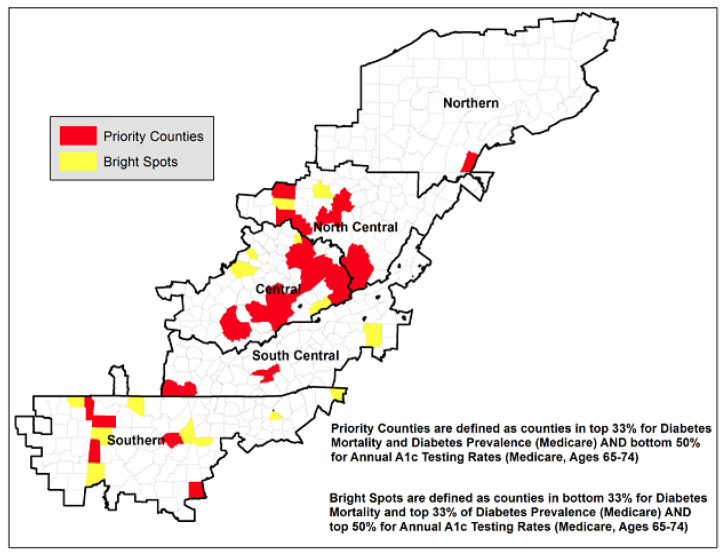
Forty-one priority counties concentrated in Central and North Central Appalachia

According to this conditional map (Figure 1), the highest rates of T2DM mortality and prevalence are concentrated in the Central and North Central Appalachian Regions (eastern KY and WV) and Southern Appalachia (MS). Low HbA1c testing rates are scattered throughout the Appalachian Region, although a large cluster of counties with low rates can be found in Northern Appalachia (Pennsylvania and New York), with other clusters of low-rate counties located in Central and North Central Appalachia. Figure 2 displays 41 priority counties (those with high T2DM mortality, high T2DM prevalence, and low HbA1c testing rates), which are concentrated in Central and North Central Appalachia. The 17 bright-spot counties (high T2DM prevalence, low T2DM mortality, and high HbA1c testing rates) are scattered throughout the Appalachian Region (apart from Northern Appalachia). Eight of the 17 bright-spot counties are adjacent to priority counties.

As expected, priority counties perform worse on diabetes measures relative to bright-spot counties and the Appalachian Region, including having higher rates of T2DM mortality and prevalence and lower HbA1c testing rates. Priority counties are also more likely to be distressed (high unemployment and poverty, and low per-capita income) and rural. Bright-spot counties have higher rates of T2DM prevalence and lower rates of T2DM mortality than the Appalachian Region, while also having lower rates of poverty and higher percentages of nonwhite populations than the region.

## IMPLICATIONS

The use of a simple, systematic framework has been explored to identify priority and bright spots in the Appalachian Region. By employing conditional mapping to T2DM multiple variables can be summarized into a single, easily interpretable map. This could be valuable for state and local health departments and T2DM-prevention programs, as they play a key role in the prevention and management of T2DM in the Appalachian Region. For example, four of the priority counties in the southern portion of the Appalachian Region are adjacent to bright-spot counties. These four counties are located in Mississippi and therefore have access to the federally funded Mississippi Diabetes Prevention and Control Program. There may be one or more programs or activities in the bright-spot counties that can be transferred to the adjacent priority counties to improve T2DM outcomes. Future research opportunities include refinement of the criteria defining priority and bright-spot counties with data on T2DM complications such as amputation rates and applying conditional mapping approaches to other health measures. Further, conducting in-depth, field investigations is necessary to understand the factors that may contribute to success in the bright-spot counties and can be replicated in other communities.^2^ A limitation of our study was using the Medicare population, and our results may not be generalizable for the non-Medicare population in the Appalachian Region.

**SUMMARY BOX**

**What is already known about this topic?** Counties in the Appalachian Region have higher rates of Type 2 diabetes mellitus (T2DM) than non-Appalachian counties, although significant geographic variation exists within the Appalachian Region.

**What is added by this report?** This research illustrates a conditional mapping approach for identifying priority and high-performing diabetes preventive care counties.

**What are the implications for public health practice, policy, and research?** This approach can be used as a starting point for in-depth research into successful strategies for improving diabetes preventive care.
